# Characterisation of magnetic atomic and molecular beamlines for the extraction of empirical scattering-matrices†

**DOI:** 10.1039/d4cp01785d

**Published:** 2024-06-11

**Authors:** Helen Chadwick

**Affiliations:** a Department of Chemistry, Faculty of Science and Engineering, Swansea University Swansea SA2 8PP UK h.j.chadwick@swansea.ac.uk

## Abstract

A recently developed magnetic molecular interferometry technique allows the experimental determination of how the amplitudes and phases of the molecular wave-function change during the collision of a gas phase molecule with a surface. This information, quantified by a scattering-matrix, provides a very stringent benchmark for developing accurate theoretical models as they can also be determined from scattering calculations and are particularly sensitive to the underlying interaction potential. However, the value of this comparison is necessarily limited by the accuracy with which an empirical scattering-matrix can be extracted from the experimental data. This paper presents the methods used to analyse the measurements and uses simulations to determine how various uncertainties in modelling the different magnetic elements which make up the beamline of the apparatus affect the accuracy with which the scattering-matrix can be extracted. It is shown that when signals have a noise level which corresponds to on the order of 1% of the oscillation amplitude, the uncertainties in the modelling do not significantly affect the ability to extract the scattering-matrix elements, with the error in the extracted values increasing to a few percent as the noise in the signals is increased to 10% of the oscillation amplitude. This therefore gives an estimate of the accuracy of the parameters that can be obtained from future measurements.

## Abstract 2

A combination of calibration experiments and modelling of the magnetic manipulation interferometer beamline allows empirical scattering matrices to be reliably extracted from the oscillation curves measured for hydrogen scattering from a surface.

## Graphical abstract

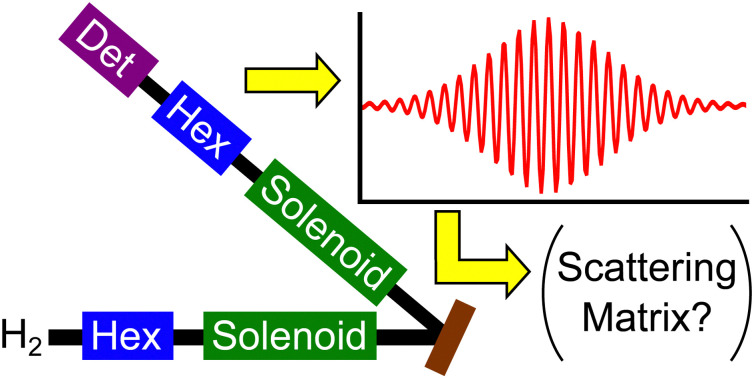

## Introduction

The interaction of H_2_ with a surface plays a key role in a range of processes, from the industrial manufacture of chemicals including ammonia through the Haber process, to the safe storage of rocket fuel and formation of stars.^1–3^ Despite being the simplest diatomic molecule, it is still not possible to model it's interaction with surfaces exactly, meaning approximations have to be made, the validity of which then have to be tested.^4–6^ One method for benchmarking these approximations is to compare the results of calculations with those from carefully controlled quantum-state resolved gas-surface scattering experiments. These experiments are particularly valuable as there are many factors that can affect the outcome of a collision with a surface, for example the molecule's translational,^7^ vibrational^8,9^ and rotational state,^10^ its orientation with respect to the surface,^11,12^ the surface temperature^13^ and surface plane.^14,15^ The observables in these measurements are typically either reaction probabilities, which characterise the reactive channel of the underlying potential energy surface (PES), or elastic or inelastic scattering probabilities, which characterise the non-reactive channel.

Using these measurements has led to great strides being made in the development of accurate theoretical models of some aspects of H_2_ scattering from surfaces, for example, in the case of the dissociation of H_2_ on Cu(111),^16^ Cu(100),^17^ Cu(110),^18^ Pt(111),^19^ Pt(211),^19^ Ru(0001),^20^ Ni(111)^21^ and NiAl(110)^22^ surfaces, the initial sticking coefficients determined experimentally can be reproduced theoretically within an accuracy of 4.2 kJ mol^−1^, often referred to as within chemical accuracy.^16–21^ There have also been numerous studies of diffractive scattering of H_2_ from surfaces^4,5,23,24^ which have provided stringent tests of theory, some of which have been met and some of which have yet to be reproduced as accurately by the calculations. For example, the PES which describes the dissociation of H_2_ on Ru(0001) with chemical accuracy fails to reproduce the diffraction intensity for elastically scattered H_2_ molecules.^20^ For H_2_ scattering from NiAl(110), the PES which accurately reproduces the dissociation probabilities^22^ also correctly predicts rotationally elastic diffraction intensities,^25^ but fails to reproduce the rotationally inelastic diffraction intensities with the same level of accuracy.^26^

Whilst the scattering studies mentioned above have provided useful benchmarks for comparing and developing accurate theoretical models, the experimental observables are only sensitive to certain aspects of the scattering-matrix (*S*-matrix), which is what theoretical models can calculate.^27^ These calculated *S*-matrices characterise how the amplitude and phase of the wave-function of the molecules before collision change due to scattering from the surface and are extremely sensitive to the underlying PES. The use of the magnetic molecular interferometer (MMI) technique allows extraction of both the relative amplitudes and phases of (rotational orientation projection) *m*_*J*_ state to *m*_*J*_ state resolved *S*-matrix elements from the experimental data, as has been demonstrated previously for the elastic scattering of H_2_ from LiF^28^ and Cu(511)^29^ surfaces, providing results which are directly comparable to those obtained from calculations. However, the value of these benchmarks is limited by how accurately the *S*-matrix can be extracted from the experimental data, which in turn is limited by how well the MMI apparatus has been characterised. This paper will present the analytical methods used to extract empirical scattering-matrices from the MMI measurements, and how any uncertainties in these methods influence the parameters that are obtained from the fits. Firstly, how signals are simulated for an MMI experiment will be discussed and how this allows scattering-matrix parameters to be extracted from the data, before addressing how uncertainties in the transmission probabilities of the different states that make up the molecular beam through two magnetic hexapole lenses in the machine affect the ability to extract the *S*-matrix parameters from the data. In this section, the influence that the signal to noise ratio of the signal which is being fit has on the accuracy of the *S*-matrix elements that are obtained from the data analysis will also be considered. Then, the methods used for obtaining the magnetic field profile through the beamline is presented and the influence that any errors in this have on the ability to analyse the data is discussed, before presenting a summary and the main conclusions of the work.

## Extraction of the scattering-matrix

**Fig. 1:**
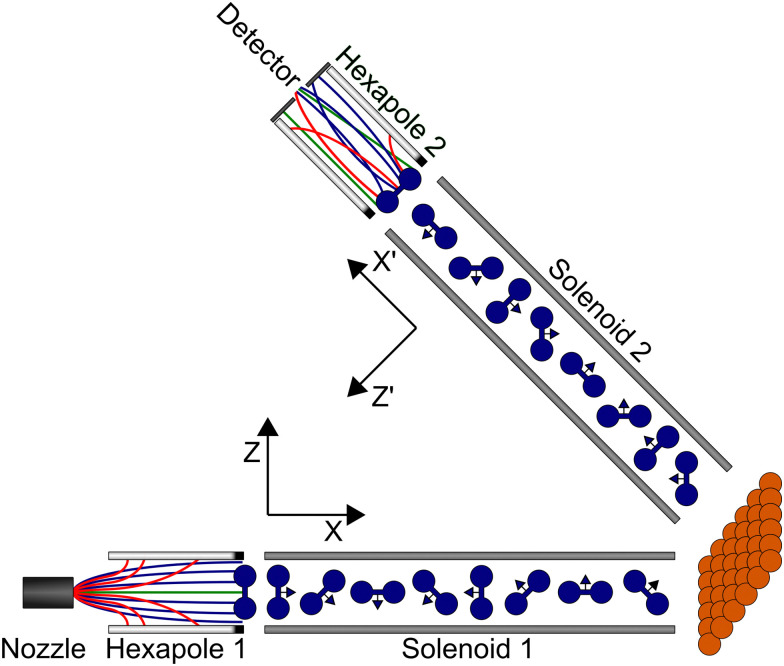
Schematic overview of the experimental apparatus used to perform magnetic molecular interferometer measurements which can be analysed to obtain a scattering-matrix and the different frames of reference used at various points in the propagation, as described in the text.

An overview of the experimental apparatus used to perform MMI measurements is shown in Fig. 1. The experimental setup has been described previously,^28–34^ and so only the main details will be presented here with a particular focus on how the different components are simulated which allows signals to be calculated and the *S*-matrix to be extracted from the data. Here, elastic scattering of H_2_ in the *I* = 1, *J* = 1 state is considered, which in the presence of a magnetic field can be split into three nuclear spin projection states (*m*_*I*_) which each split into three rotational orientation projection states (*m*_*J*_), giving a total of nine *m*_*I*_, *m*_*J*_ states, the energies of which can be calculated using the Hamiltonian defined by Ramsey.^35^

A nozzle is used to generate the molecular beam expansion, where it is assumed that all nine *m*_*I*_*, m*_*J*_ states of hydrogen in *I* = 1, *J* = 1 are equally populated. This will also contain molecules in *I* = 0, *J* = 0 which cannot be manipulated in the apparatus, and which give a constant background in the measurements. The central part of the expansion passes through a skimmer to create a molecular beam which then continues through the rest of the beamline of the apparatus. There are two hexapoles^36,37^ in the beamline, which create inhomogeneous magnetic fields where the different *m*_*I*_, *m*_*J*_ states experience either a restoring or deflecting force depending on whether their magnetic moment is greater than or less than 0, and two solenoids, which create tuneable uniform magnetic fields which allow the coherent control of the *m*_*I*_, *m*_*J*_ states as characterised by the Hamiltonian given by Ramsey.^35^ At the end of the first arm of the machine, there is a UHV chamber where the surface is mounted, and at the end of the beamline, there is a highly sensitive custom built detector^38^ which operates on the same principle as a mass spectrometer, ionising the molecules, mass filtering them and detecting the resulting current.

In previous studies^28,30,32,34^ the probabilities that the different *m*_*I*_, *m*_*J*_ states are transmitted through the second hexapole were approximated as being independent of the probability that they are transmitted through the first, which gave 18 hexapole probabilities, one each for the transmission of the nine initial *m*_*I*_, *m*_*J*_ states through the first hexapole, and another nine for the transmission of each of these states through the second hexapole. However, the probability that an *m*_*I*_, *m*_*J*_ state will be transmitted through the second hexapole will depend both on its magnetic moment and its trajectory through the machine, which is influenced by the first hexapole.^39^ For example, a molecule which is initially in the *m*_*I*_ = 0, *m*_*J*_ = 0 state will not be deflected by the first hexapole and therefore follow a slightly divergent path, but one in the *m*_*I*_ = −1, *m*_*J*_ = −1 state will be focussed to follow a parallel path through the machine. If both molecules reach the entrance of the second hexapole in the *m*_*I*_ = −1, *m*_*J*_ = −1 state, then the probability of the second hexapole transmitting them will be different despite them being in the same final *m*_*I*_, *m*_*J*_ state. To account for this, the semi-classical trajectory code used previously to calculate the hexapole probabilities^40^ for both arms of the machine independently has been modified to allow the trajectories to undergo either specular or diffractive scattering at the surface and continue down the second arm of the machine to calculate probabilities for all 81 initial state, final state combinations. It was found that these 81 values could not be reproduced using the initial approach of modelling the two arms independently, and therefore this more sophisticated method of calculating the hexapole probabilities is required. The hexapole probability for a state being transmitted through both hexapoles for an initial *m*_*I*_, *m*_*J*_ state |*n*〉 in the first hexapole and final *m*_*I*_, *m*_*J*_ state |*f*〉 in the second hexapole will be denoted as *P*_hex_(*f* ← *n*), where the quantisation axes for these states are defined by a dipole element directed along *Z* immediately after the end of the first hexapole and −*Z*′ before the start of the second.

At the end of the first hexapole, the states in the molecular beam are still described as pure *m*_*I*_, *m*_*J*_ states but with different populations, as any superposition states decohere in the strong magnetic field gradients of the hexapole.^41^ For this reason, the starting point of the coherent wave-function propagation of the molecules through the measured magnetic field profile of the machine are the pure *m*_*I*_, *m*_*J*_ states defined with respect to the *Z* axis (see Fig. 1). Each pure *m*_*I*_, *m*_*J*_ state is propagated through the magnetic field profile of the first arm of the machine up to the surface using the Ramsey Hamiltonian^35^ using the methods described in ref. 30. Repeating this calculation for each of the nine pure *m*_*I*_, *m*_*J*_ states produces the coherent super-position states that the initial states evolve into, which is denoted as *U*(*B*_1_). The calculation is performed in the same way for propagating the molecules through the second arm of the machine, although the quantisation axis is taken to be the *Z*′ direction (see Fig. 1). The resulting coherent super-position states obtained from propagating the nine pure *m*_*I*_*, m*_*J*_ states through the second arm are denoted as *U*(*B*_2_).

After passing through the first hexapole and first solenoid, the molecules scatter from the surface, where it is assumed that the *m*_*I*_ state is a spectator to the collision as the surfaces considered here are non-magnetic, and the interaction time too short for spin–rotation or spin–spin couplings to significantly alter the state of the molecule. The scattering process can change both the amplitude and phase of the (*m*_*J*_ state) wave-functions, which is characterised by the scattering-matrix (*S*). Multiplying the wave-function before scattering by the scattering-matrix gives the wave-function after scattering. For the case of the elastic scattering of H_2_ in *I* = 1, *J* = 1, *S* is a 3 × 3 matrix of complex numbers where each element is related to the scattering of an initial *m*_*J*_ state (*n*′) to a final *m*_*J*_ state (*f*′), where the quantisation axis is taken to be the surface normal. Note that the quantisation axis chosen for calculating the scattering-matrix is arbitrary in the sense that it will not affect the outcome of the scattering event and therefore the surface normal was chosen to follow previous conventions.^42^ In principle, this matrix contains 18 parameters in total, as it contains 9 amplitudes (
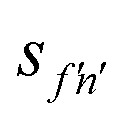
) and 9 phases (
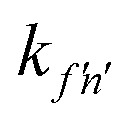
) as given in eqn (1).1
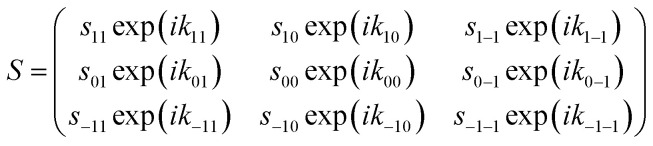
Here, scattering processes are considered where the surface has reflection symmetry in the scattering plane which places restrictions on the amplitudes and phases of the scattering-matrix elements^31^ which can be attributed to the scattering process being unable to distinguish between molecules that are in the *m*_*J*_ = 1 and *m*_*J*_ = −1 states. This reduces the number of parameters to 10 and means the *S*-matrix can be written as in eqn (2).2
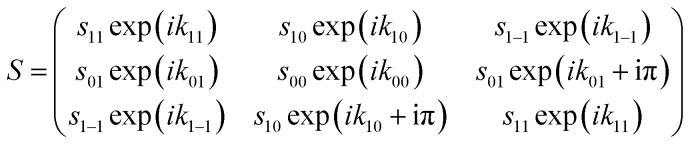
Additionally, as the analysis is only sensitive to the relative size of the oscillation due to uncertainties with determining the background and fraction of molecules that do not follow the magnetic manipulation in the apparatus, only relative *S*-matrix elements can be extracted from the data. In effect, this reduces the number of unique parameters that describe the *S*-matrix to 8, as the values that are obtained are normalised with respect to the element with the largest amplitude.

Combining the above, it is possible to write the coherent-superposition state (|*Ψ*_*n*_〉) resulting from the propagation of an initial pure *m*_*I*_, *m*_*J*_ state |*n*〉 from the end of the first hexapole to the start of the second as

3 |*Ψ*_*n*_〉 = *U*(*B*_2_)*R*(*θ*_2_)*SR*(*θ*_1_)*U*(*B*_1_)|*n*〉

The two rotation matrices included in eqn (3) change the quantisation axis from *Z* to the surface normal (*R*(*θ*_1_)), and from the surface normal to *Z*′ (*R*(*θ*_2_)). It follows that the signal that would be obtained for a molecule travelling with a single velocity *v* in the initial state |*n*〉 scattering to the final state |*f*〉 would be proportional to4
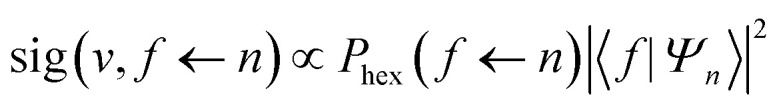
However, all nine initial *m*_*I*_, *m*_*J*_ states and all nine final *m*_*I*_, *m*_*J*_ states contribute to the experimental signal. Additionally, there are a range of velocities in the molecular beam expansion, with different weights (*P*_*v*_). Therefore it is necessary to sum the individual signals defined by eqn (4) over all the initial states, final states and the weighted velocity distribution of the molecular beam to give the total measured signal as5
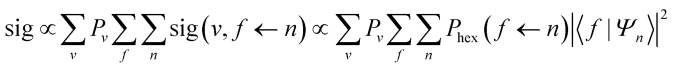
It follows that the only unknown in the signal expression given by eqn (5) is the scattering-matrix, which can be extracted from the experimental data as shown previously.^28,29^ These empirical scattering-matrices are directly comparable to those obtained from state-of-the-art theoretical models^42^ which is why the MMI experiments provide such valuable benchmarks for these calculations; both the amplitudes and phases of the *S*-matrix elements can be obtained, whereas other measurements, for example which measure scattering or reaction probabilities,^43–45^ determine quantities which are equal to the square modulus of *S*-matrix elements. Whilst these do still have great value and play an important role in testing theoretical models,^4,6,46,47^ they are insensitive to the phases of the *S*-matrix elements and are often also summed over more than one element of the *S*-matrix. However, these complex *S*-matrix elements can only be extracted with an accuracy which is determined by how well the hexapole probabilities and magnetic field profiles of the two arms of the machine have been characterised, as well as how accurately the MMI signal has been measured. These will be addressed below, focussing on how significant an effect these uncertainties have on the empirical *S*-matrix elements obtained from the measurement.

## Hexapole probabilities

**Fig. 2:**
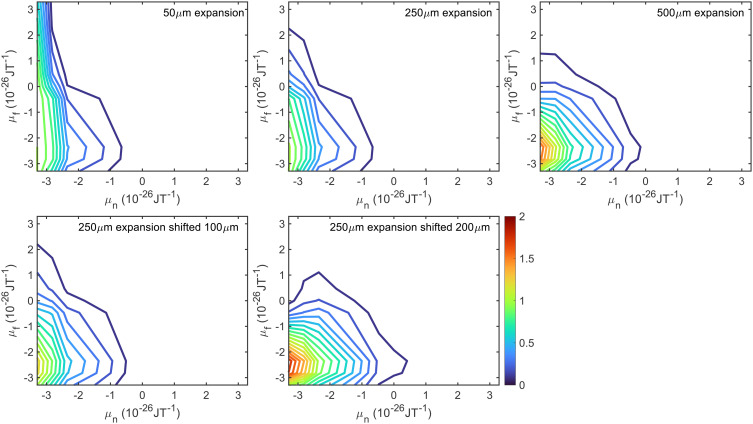
Hexapole probabilities calculated using the trajectory calculations described in the text for H_2_ travelling at a velocity of 1470 m s^−1^ for a 50 μm expansion (top left), 250 μm expansion (top middle), 500 μm expansion (top right), 250 μm expansion shifted by 100 μm (bottom left) and 250 μm expansion shifted by 200 μm (bottom middle).

The first uncertainty in the signal calculation is related to the size of the molecular beam source which creates the supersonic expansion. The diameter of the hole in the nozzle is on the order of 50 μm, and the skimmer which selects the central part of the beam is 500 μm. However, due to the high density within the skimmer, it is possible that the skimmer acts as a source to a secondary expansion,^48^ or that the apparent aperture size which creates the expansion is somewhere in between the two. Therefore, trajectory calculations were performed using an initial beam size of 50 μm (top left), 250 μm (top middle) and 500 μm (top right), the results of which are presented in the top row of Fig. 2, where they have been normalised so that the probability for transmitting the molecules in the initial and final *m*_*I*_ = −1, *m*_*J*_ = −1 state is 1. An intermediate value of 250 μm was chosen as well as the two extremes as the beam profile calculated using this size source most closely matches measurements of the profile at the position of the surface. In all cases, only molecules with a dipole moment of less than zero in the first arm (*μ*_*n*_ < 0) have any significant probability of being transmitted and contributing to the signal. As the source size is increased, the probability of the defocussed states in the second hexapole (*μ*_*f*_ > 0) contributing to the signal decreases, the reason for this is that a beam produced by a larger nozzle is characterised by a larger spot illuminating the sample and a larger deflection of trajectories, both of which increase the *μ*_*f*_ < 0 selectivity of the second hexapole.

A second uncertainty is due to the nozzle position with respect to the entrance of the first hexapole. Whilst before experimental measurements the nozzle position is optimised to maximise the pressure of the molecular beam going into the UHV chamber, this does not exclude the possibility that the nozzle is slightly misaligned. Hexapole probabilities were therefore also calculated using a 250 μm expansion source with a 100 μm misalignment (bottom left) and with a 200 μm misalignment (bottom right), with the results shown as the contour plots in the bottom row of Fig. 2. The 200 μm shift has a larger effect on the probabilities, with the most significant differences again being seen for *μ*_*f*_ > 0.

**Fig. 3:**
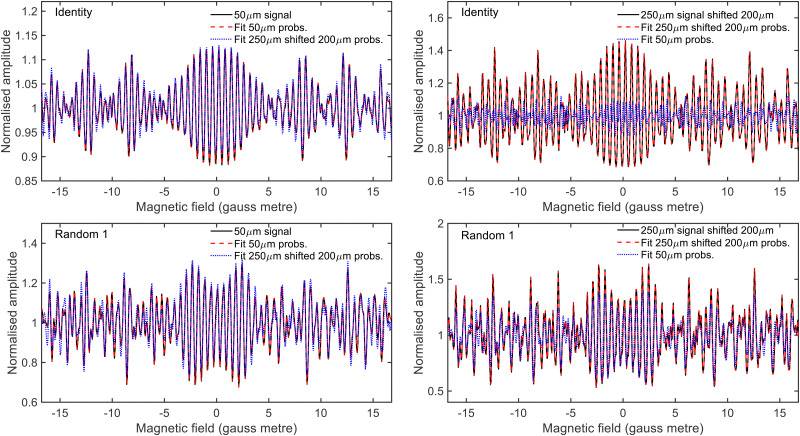
Fits to simulated signals generated using the identity scattering-matrix (top row) and a randomly generated scattering-matrix (bottom row) using the 50 μm expansion probabilities (left column) and the 250 μm expansion shifted by 200 μm probabilities (right column). The simulated signals are shown in black, the fit obtained with the ‘correct’ hexapole probabilities as a red dashed line, and with the ‘incorrect’ hexapole probabilities as a blue dotted line (see text for details). Only the fits to the *B*_2_ = 0 gauss metre simulations are presented here.

To determine the effect that the uncertainty in the hexapole probabilities has on being able to fit experimental data, signals were simulated for scanning *B*_1_ at *B*_2_ values of 0 gauss metre and 11.2 gauss metre using an identity scattering-matrix and three randomly generated scattering-matrices (within the constraints given in eqn (2)) and the fitted hexapole probabilities corresponding to a 50 μm expansion and to a 250 μm source with a 200 μm shift. These signals were then analysed in the same way as used to analyse previous experimental data,^28,31^*i.e.*, allowing the *S*-matrix parameters and a background parameter to vary. The fits were run 100 times with initially randomised parameters using the Nelder and Mead downhill simplex algorithm^49^ with simulated annealing to minimise the difference between the input signals and fits, and the results that produced the minimum error are presented in Fig. 3, where the top row corresponds to simulated signals generated using the identity scattering-matrix and the bottom row the first randomly generated scattering-matrix, and the left and right columns correspond to signals which were simulated with 50 μm expansion probabilities and signals simulated with 250 μm expansion shifted by 200 μm probabilities respectively. In each panel, the black line shows the initial simulated signal that was fit to, the red dashed line the fit to the data that was obtained using the hexapole probabilities that were used to produce the simulated data and the blue dotted line the fit to the data obtained by using the other set of hexapole probabilities. As can be seen, it is not possible to fit the simulated signals with the incorrect hexapole probabilities and the sensitivity to the probability values used varies for different scattering-matrices. As a result, this uncertainty needs to be incorporated into the method used to analyse the experimental data.

Whilst all 81 hexapole probabilities could be allowed to vary in the signal analysis, at least in principle, it is unlikely that the fits will converge on a single set of parameters. Furthermore, many of these probabilities are very low and have a negligible effect on the signal. It is therefore desirable to try and find a reduced parameter model which describes these probabilities, the parameters for which could then be freed when analysing the experimental data. The model that was used was a double Gaussian distribution, given by

6 *P*_hex_(*f* ← *n*) = *A* exp(−*σ*_1_(*μ*_*n*_ − *μ*_1_)^2^ − (*Cμ*_*n*_ + *σ*_2_)(*μ*_*f*_ − *μ*_2_)^2^)

where *A* is a (arbitrary) normalisation factor, *σ*_*x*_ parameterises the width and *μ*_*x*_ the peak position of the Gaussian distribution where *x* = 1 and 2 for the first and second arm respectively, and *C* is a parameter which allows the width of the Gaussian for the second arm to be dependent on the dipole moment of the state in the first arm. *μ*_*n*_ and *μ*_*f*_ are the magnetic dipole moments of the initial and final *m*_*I*_, *m*_*J*_ state and are calculated using

**Fig. 4:**
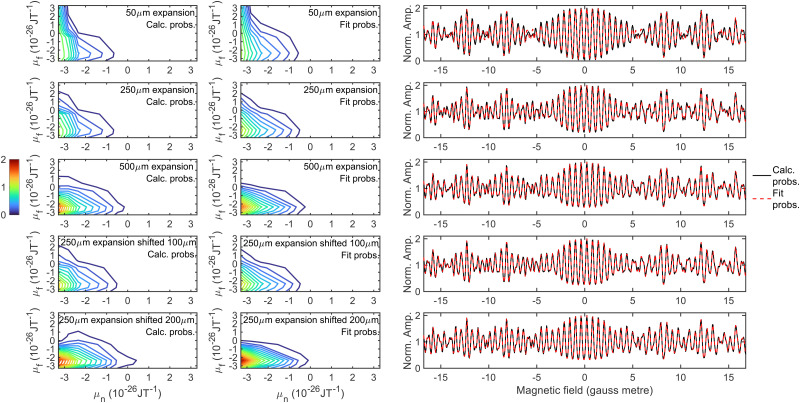
Hexapole probabilities calculated using the trajectory calculations described in the text (left column), the fit to the probabilities obtained using the parameters in Table 1 in eqn (6) (middle column), and a comparison of the simulated signals obtained using the calculated (black solid line) and fit (red dashed line) hexapole probabilities. The calculations were performed for a 50 μm expansion (top row), 250 μm expansion without a shift (second row), 500 μm expansion (third row), 250 μm expansion with a 100 μm shift (fourth row) and 250 μm expansion 200 μm shift (bottom row).

7 *μ*_*y*_ = −*am*_*I*_ − *bm*_*J*_

where *y* corresponds to either *n* or *f*, and *a* = 2.82 × 10^−26^ J T^−1^ and *b* = 0.45 × 10^−26^ J T^−1^ to the nuclear and rotational dipole moment of H_2_^35^ respectively. When fitting the hexapole probabilities for the five different expansions presented in Fig. 2, the values of *σ*_1_, *μ*_1_ and *σ*_2_ were constrained to be the same for each set of probabilities, with *C* and *μ*_2_ allowed to vary. The fits to the hexapole probabilities that were obtained are presented in the middle column of Fig. 4, which can be compared to the values obtained from the trajectory calculations presented in the first column for the different expansion conditions. The values of the parameters used in eqn (6) to obtain these fits are given in Table 1. Whilst there are differences in the contour plots (most noticeably in the case of the 250 μm expansion with a 200 μm shift in the bottom row of the figure), the calculated signals in the right column of Fig. 4 show that these differences do not significantly affect the calculated signals, with the black lines showing the signals simulated using the calculated hexapole probabilities presented in the left column, and the red dashed lines those calculated with the fit probabilities in the middle column, although there are some very small differences around ±6 gauss metre, ±11 gauss metre and ±14 gauss metre in the case of the 50 μm expansion in the top right panel. Whilst the result here is presented for the identity scattering-matrix, the same has been determined to be true for 100 randomly generated scattering-matrices. This suggests that the uncertainty in the nozzle expansion size and position can be accounted for by two parameters in the calculation of the hexapole probabilities.

**Table 1:** Parameters obtained from fitting the hexapole probabilities presented in Fig. 2 using eqn (6) where the values of *σ*_1_, *μ*_1_ and *σ*_2_ were constrained to be the same in each case, and *C* and *μ*_2_ were allowed to vary

Set of probabilities	Parameter				
	*σ*_1_	*μ*_1_	*C*	*σ*_2_	*μ*_2_
50 μm	0.0967	−6.5482	0.1763	0.5966	−2.1123
250 μm no shift	0.0967	−6.5482	0.1367	0.5966	−2.2900
500 μm	0.0967	−6.5482	0.0604	0.5966	−2.1770
250 μm 100 μm shift	0.0967	−6.5482	0.1186	0.5966	−2.2517
250 μm 200 μm shift	0.0967	−6.5482	0.0000	0.5966	−2.2284

**Fig. 5:**
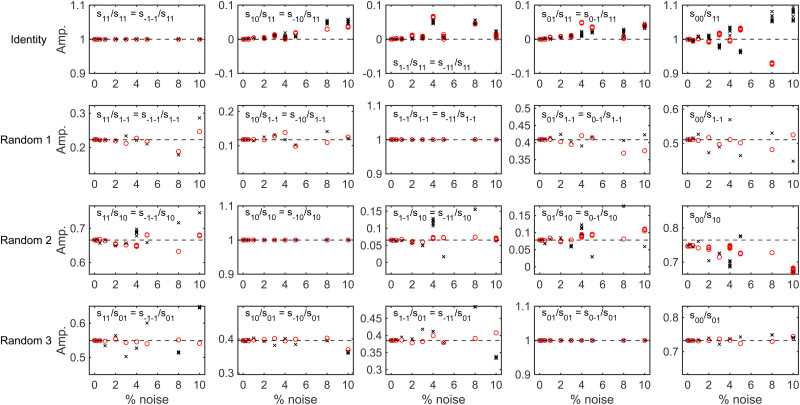
The (unique) relative amplitudes of the scattering-matrix elements obtained by fitting the signals generated for an identity scattering-matrix (top row), the first randomly generated scattering-matrix (second row), the second randomly generated scattering-matrix (third row) and the third randomly generated scattering-matrix (bottom row) with varying noise levels using the 50 μm expansion hexapole probabilities (black crosses) and the 250 μm shifted by 200 μm expansion (red circles). The dashed lines show the value of the parameter used to simulate the signal.

**Fig. 6:**
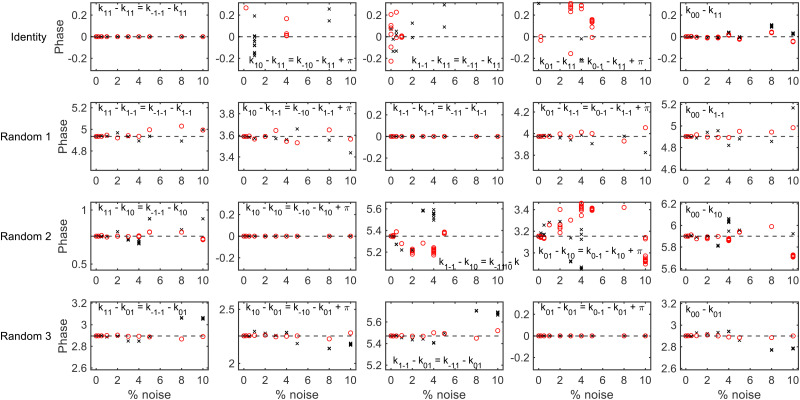
The (unique) relative phases of the scattering-matrix elements obtained by fitting the signals generated for an identity scattering-matrix (top row), the first randomly generated scattering-matrix (second row), the second randomly generated scattering-matrix (third row) and the third randomly generated scattering-matrix (bottom row) with varying noise levels using the 50 μm expansion hexapole probabilities (black crosses) and the 250 μm shifted by 200 μm expansion (red circles). The dashed lines show the value of the parameter used to simulate the signal.

To determine whether it is possible to account for the uncertainty in the expansion size and nozzle position when analysing the experimental data whilst still being able to reliably extract a scattering-matrix, fits were run on simulated signals generated using the identity scattering-matrix and three randomly generated scattering-matrices using the hexapole probabilities corresponding to a 50 μm expansion and a 250 μm expansion shifted by 200 μm. Before running the fits, a constant background was added to each signal to mimic the polarisation losses that occur through the beamline as well as random noise sampled from a Gaussian distribution with a standard deviation defined as a percentage of the oscillation amplitude (maximum–minimum). When fitting the data the scattering-matrix elements, a constant to account for the background and the values of *C* and *μ*_2_ in eqn (6) were allowed to vary, with the latter two parameters limited to the range that cover the values in Table 1. The relative scattering-matrix amplitudes and phases obtained from the analysis of the signal generated using the identity scattering-matrix (top row), first random scattering-matrix (second row), second random scattering-matrix (third row) and third random scattering-matrix (bottom row) are shown in Fig. 5 and 6 respectively, where each panel corresponds to the *S*-matrix element given in the label, and only the unique values from eqn (2) are presented. The results corresponding to the signal generated using the 50 μm expansion hexapole probabilities are shown as black crosses, the 250 μm expansion shifted by 200 μm hexapole probabilities as red circles, and the dashed lines the values of the (relative) scattering-matrix parameter which was used to simulate the signal. In each panel the results from the best 10 fits to each set of data are presented, as it has previously been demonstrated^29^ that considering only a small number of fits with the lowest fitting error is a valid way of presenting the results and has no effect on the conclusions. At low noise levels, the returned *S*-matrix elements correspond to those that were used to calculate the data for signals simulated with both sets of hexapole probabilities, demonstrating that the uncertainty in the hexapole probabilities can be accounted for when fitting the data and does not affect the ability to extract the scattering-matrix elements. As expected, the values of the *S*-matrix elements obtained from the fits deviate more significantly from the value used to simulate the data as the noise level increases. The results that are presented also suggest that if the noise level is on the order of 1%, the values of the scattering-matrix parameters that are obtained from the fit are likely to be accurate. In some cases, most notably the middle three panels in the top row of Fig. 6 and the third and fourth panels in the third row of Fig. 6, the errors in the phases are larger, going beyond the ±0.314 limits of the axes (*i.e.*, ±5% of the maximum range of values). In all cases, these phases correspond to values of *S*-matrix amplitudes which are zero (in the case of the identity scattering-matrix in the top row) or small values of *S*-matrix amplitudes (in the case of the second random scattering-matrix in the third row; see the equivalent panels in Fig. 5) meaning these elements do not significantly contribute to the signal. Consequently, there is a larger uncertainty in the value of these phases, even at lower noise levels. Whilst the extracted *S*-matrix parameters do become less accurate as the noise increases, the deviation between the value input to the simulation and value obtained from the fit tend to lie within ±0.1 in the case of the amplitudes, and ±0.314 in the case of the phases. This means that even for noisier data the errors on the returned scattering-matrix parameters are likely to be on this order, although it is important to note that if the *S*-matrix amplitudes are small, this will likely lead to larger errors in the associated phases as discussed above.

It is also worth noting that if the experimental data is too noisy to reliably extract an *S*-matrix, it could still be used to benchmark theoretical models as a signal could be simulated using the *S*-matrix obtained from the calculation and compared to the experimental data, mirroring an approach used previously when studying rotationally inelastic scattering^31^ where it was not possible to do the two different measurements^28^ that are required to extract a unique scattering-matrix. If the measured and simulated signals disagree the result would suggest the model is not correct, and if the two agree it would lend support that the model could be correct, although it is unlikely to provide definitive proof of the accuracy as many signals could agree with the measurement within the error bars of the data.

**Fig. 7:**
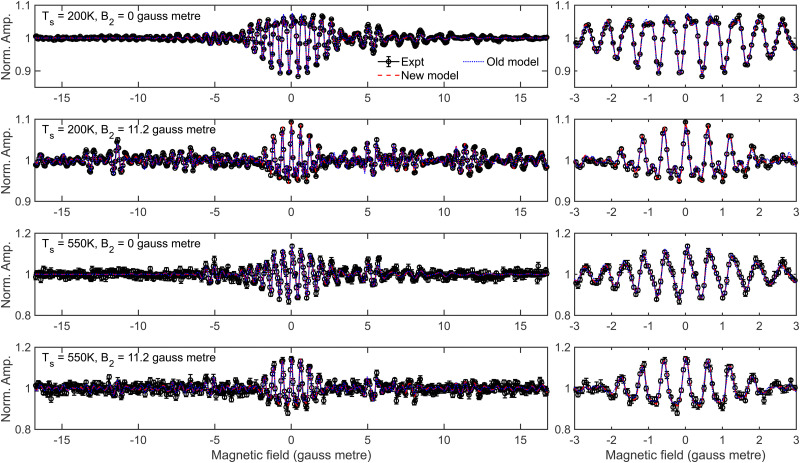
Fits to the measured oscillation curves (black) for the specular scattering of H_2_ from Cu(511) obtained using the new model for the hexapole probabilities (red dashed line) and old model (blue dotted line) at a surface temperature of 200 K for *B*_2_ = 0 gauss metre (top row) and *B*_2_ = 11.2 gauss metre (second row), and at a surface temperature of 550 K for *B*_2_ = 0 gauss metre (third row) and *B*_2_ = 11.2 gauss metre (fourth row). The right column is a magnification of the central part of the signal presented in the left column.

To conclude this section, a comparison of the results obtained by fitting data using the method described above in which the transmission probabilities of the molecules through the second hexapole depends on their *m*_*I*_, *m*_*J*_ state in the first hexapole (which will be described in the following as the new model), and the method of having independent hexapole probabilities characterising the transmission probabilities through the first and second arm of the machine used in previous studies^28,32^ (referred to as the old model) is presented. Recently published data taken for the specular scattering of H_2_ from Cu(511) at different surface temperatures^29^ will be used as an example, where the analysis in that work was performed using the new model. Fig. 7 presents the fits obtained to the measurement (black line) using the new model (red dashed line) and the old model (blue dotted line) for a *B*_1_ scan at *B*_2_ = 0 gauss metre (top row) and a *B*_1_ scan at *B*_2_ = 11.2 gauss metre (second row) for a surface temperature of 200 K, with the equivalent data measured at a surface temperature of 550 K being shown in the third and fourth rows. The left column presents all the data, whereas the right column shows a magnification of the central oscillation. The fits to the data in both cases are very good, but as can be seen the fit to the data using the new model tends to be slightly better than that of using the old model. In ref. 29 it was concluded that the rotational selectivity and rotational polarisation (see eqn (7) and (8) in ref. 29 for the definition of these quantities) decreased as the surface temperature increased. Table 2 presents the values of these parameters at the two surface temperatures obtained from the fits to the measured data using the two different hexapole probability models. As can be seen, the trends in the rotational selectivity and rotational polarisation are the same regardless of which model is used to describe the hexapole probabilities when analysing the data, *i.e.*, both decrease as the surface temperature is increased, meaning had the old model been used it would not have affected the conclusions of that work.

**Table 2:** The rotational selectivity and rotational polarisation obtained using the old model and the new model presented here for the hexapole probabilities for the specular scattering of H_2_ from a Cu(511) surface at a temperature of 200 K and 550 K. The results for the new model are taken from ref. 29

Model	Surface temperature = 200 K		Surface temperature = 550 K	
	Selectivity	Polarisation	Selectivity	Polarisation
Old model	0.48	0.62	0.29	0.43
New model^29^	0.49	0.56	0.35	0.39

## Magnetic field profiles

**Fig. 8:**
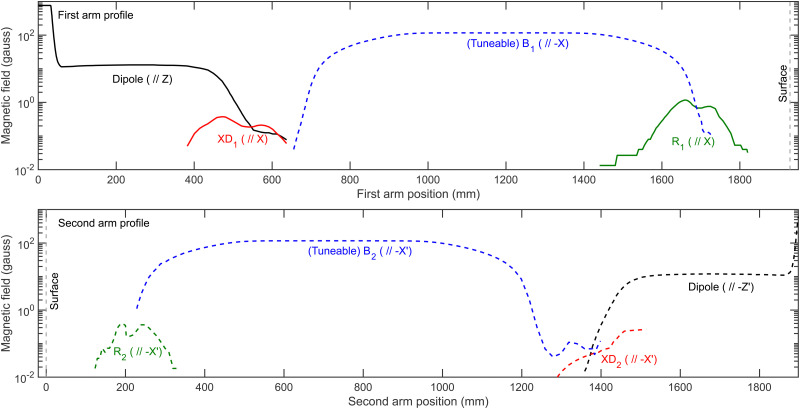
Measured magnetic field profile for the first arm (top panel) and second arm (bottom panel) of the MMI beamline showing the positions of the dipole fields which define the quantisation axes (black), the (tuneable) solenoid fields (blue), the residual fields (green) and the *X* (*X*′) components of the dipole fields (red). Solid lines correspond to positive magnetic fields, and dashed lines to negative magnetic fields (note the log scale used for the *y*-axis).

To calculate an MMI signal accurately, it is also necessary to have characterised the magnetic field profile of the beamline through which the coherent evolution of the *m*_*I*_, *m*_*J*_ states are calculated using the Ramsey Hamiltonian.^35^ Measurements of the magnetic field profile have been performed^39^ and the results are presented in the top and bottom panels of Fig. 8 for the first and second arm of the beamline respectively, where solid lines correspond to positive magnetic field values and dashed lines to negative magnetic field values, with the grey vertical dashed line showing the position of the surface in each profile. The profiles consist of dipole fields that define the quantisation axes for the initial (along *Z*, black line, top panel) and final (along −*Z*′, dashed black line, bottom panel) state selection, and solenoid fields which cause the coherent evolution of the wavefunction before (*B*_1_, along −*X*, dashed blue line, top panel) and after (*B*_2_, along −*X*′, dashed blue line, bottom panel) scattering from the surface. Additionally, there are small fields which are directed along *X* at the end of the first dipole field (XD_1_, red line, top panel) and at the entrance of the scattering chamber (*R*_1_, green line, top panel), and directed along −*X*′ at the exit of the scattering chamber (*R*_2_, dashed green line, bottom panel) and at the start of the second dipole (XD_2_, dashed red line, bottom panel). *R*_1_ and *R*_2_ will be referred to as residual fields, and XD_1_, XD_2_, *R*_1_ and *R*_2_ will be referred to collectively as non-ideal fields.

The measurement of the magnetic field profile^39^ was done using two different commercially available probes, with an AlphaLab Vector Gauss Meter being used for the majority of the beamline, but a LakeShore Instruments model 410 probe where the magnetic fields are greater than 200 gauss. These have limited accuracy which will be particularly problematic when measuring the non-ideal fields present in the profile, with the AlphaLab Vector Gauss Meter having a stated accuracy of ±0.02 gauss. This error can further be compounded as the probe needs zeroing in a zero-field region just prior to the measurement (before drift effects become significant), which can introduce an additional small offset to the measurement. Whilst the probe can measure the *X*, *Y* and *Z* components of the field simultaneously, there is a small (∼mm) offset between the sensors which means that the field values measured at a particular distance along the beamline are not at the same point in space, which will also introduce a small inaccuracy. Additionally, due to the physical size of the probe it is not possible to measure the field profile across the diameter of the molecular beam path, where it would be reasonable to assume it could vary at different positions. There are also inherent challenges with these measurements which reduces their accuracy, not least that the total beamline that needs characterising is over 3 metres long and in places where the magnitude of the magnetic field changes quickly measurements are required at separations on the order of 1 mm. Inaccessibility of some parts of the beamline also presents a problem, making that region of the profile difficult to measure. Furthermore, it is also difficult to ensure that the probe is in the correct orientation, and therefore there could also be an error of several degrees in the angle, which would be expected to have a particularly marked effect on the measurement of XD_1_ and XD_2_ due to the presence of the stronger dipole field.

Whilst the measured magnetic fields throughout the apparatus provide a valuable starting point for the profile, due to the difficulties mentioned above they are not expected necessarily to be sufficiently accurate to use in the analysis of MMI measurements. A good method for determining whether this is the case is to compare measurements of ^3^He scattering from a Cu(111) surface performed at different nozzle temperatures with signals calculated using wave-functions propagated through the measured magnetic field profiles. ^3^He is used as only the initial *m*_*I*_ = 1/2 state and final *m*_*I*_ = 1/2 state can be considered to contribute to the signal (any atoms in *m*_*I*_ = −1/2 will just reduce the amplitude of the oscillation) which removes any uncertainty due to the hexapole transmission probabilities. Likewise Cu(111) is used as it is non-magnetic, and therefore the *m*_*I*_ state is not expected to change due to scattering and the *S*-matrix is therefore an identity scattering-matrix which also removes that ambiguity. This means that the small differences that are observed between the experimental and the calculated signals can be attributed to inaccuracies in the magnetic field profile. Nevertheless, these small differences can be removed by making small corrections to the non-ideal fields that are present in the measured magnetic field profile. Before describing how this can be achieved, it is first necessary to present the features of the ^3^He signals that are obtained and how they are sensitive to different aspects of the magnetic field profile.

**Fig. 9:**
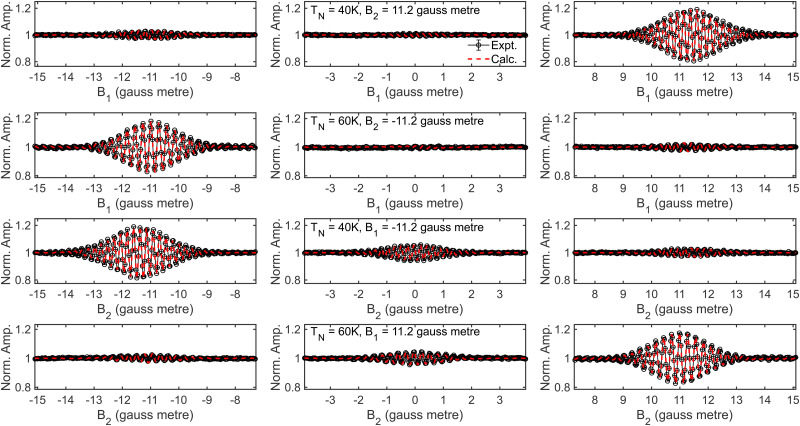
Comparison of the measured ^3^He scattering from Cu(111) signals (black solid line) with the calculated signals (red dashed line) for *B*_1_ scans when *B*_2_ = 11.2 gauss metre at a nozzle temperature of 40 K (top row), *B*_1_ scans when *B*_2_ = −11.2 gauss metre at a nozzle temperature of 60 K (second row), *B*_2_ scans when *B*_1_ = −11.2 gauss metre at a nozzle temperature of 40 K (third row) and *B*_2_ scans when *B*_1_ = 11.2 gauss metre at a nozzle temperature of 60 K (bottom row). Each column magnifies a different echo, with the first and third showing either the parallel echo (*B*_1_ = *B*_2_, the largest amplitude) or anti-parallel echo (*B*_1_ = −*B*_2_, the smallest amplitude), and the middle column showing the X′ echo in the *B*_1_ scans (top two rows) or X echo in the *B*_2_ scans (bottom two rows).

Examples of experimental signals for ^3^He scattering from Cu(111) are presented as black lines in Fig. 9, where the signal in each row was measured at the nozzle temperature and fixed field detailed in the middle panel of that row, and each column focusses on a different region of the magnetic field scan showing a different spin-echo, with the first and third column showing either the parallel^50^ (*B*_1_ = *B*_2_, largest amplitude) or anti-parallel^50^ (*B*_1_ = −*B*_2_, smallest amplitude) echo, and the middle column showing the X′^33^ echo when *B*_1_ is scanned (top two rows) and the X^33^ echo when *B*_2_ is scanned (bottom two rows). Results from simulations, which are described and presented in the ESI,† show that the amplitude and phases of each of these echoes has a different dependence on each of the non-ideal fields that are present in the measured magnetic field profile, which provides a strategy for determining the integral of these fields in the beamline as described below.

As shown in the third rows of Fig. S1 and S2 (ESI†), if the value of *R*_1_ − *R*_2_ is kept constant, the signal for the parallel echo is the same regardless of the individual values of *R*_1_ and *R*_2_. This property is used to determine the value of the difference between the residual fields by comparing the oscillations around the parallel echo (*B*_1_ = *B*_2_) in the *B*_1_ (*B*_2_) scans that were measured at non-zero values of *B*_2_ (*B*_1_) with parallel echoes simulated for different values of *R*_1_ − *R*_2_, to find the value of *R*_1_ − *R*_2_ that minimises the difference between the calculated and measured signals. Then, the same is done for the anti-parallel echo (*B*_1_ = −*B*_2_) which is shown to be independent of the individual values of *R*_1_ and *R*_2_ when the sum of the two is kept constant (fourth row, Fig. S1 and S2, ESI†), which provides the value of *R*_1_ + *R*_2_. These two results allow the values of *R*_1_ and *R*_2_ to be found. Finally, the value of XD_1_ is determined by comparing the amplitude of the simulated and measured X′ echo (fifth row, Fig. S2, ESI†), and XD_2_ optimised using the same procedure but using the X echo (sixth row, Fig. S1, ESI†). In all optimisations, the value of the field being optimised is changed by changing the integral of the fields shown in the top and bottom panels of Fig. 8 but leaving the length and shape the same as that in the measured profile.

A comparison of the experimental data (black) and signals calculated (red dashed lines) with wave-functions propagated through the optimised magnetic field profiles is shown in Fig. 9, with the two shown to be in excellent agreement in the case of these echoes. As a final check, these optimised profiles are then used to run wave-function propagations to calculate *U*(*B*_*x*_) which are then used to simulate signals for the *B*_1_ and *B*_2_ scans where *B*_2_ = 0 gauss metre and *B*_1_ = 0 gauss metre respectively to compare with measurements performed under the same conditions. The excellent agreement is also observed in this case, as shown in Fig. S4 of the ESI.† Whilst the optimised magnetic field profiles that are obtained using this method clearly result in calculated signals that reproduce the ^3^He measurements well, the values of *R*_1_ and *R*_2_ are reduced by 2 to 3 gauss centimetre, and XD_1_ and XD_2_ reduced to approximately 40% of the original measured value.

The simplicity of the spin evolution of ^3^He results in a sensitivity to the total magnetic field integrals rather than to the exact shape of the magnetic field profile, meaning that while the procedure outlined above allows the integrals of the various non-ideal fields to be determined, it cannot provide unique values for the length and magnitude of the field. However, when the optimised magnetic profiles are used for more complex beams, such as H_2_, the exact shape of the profile rather than just the field integral might be important. It is therefore important to determine to what degree the precise details of these non-ideal fields need to be known.

**Fig. 10:**
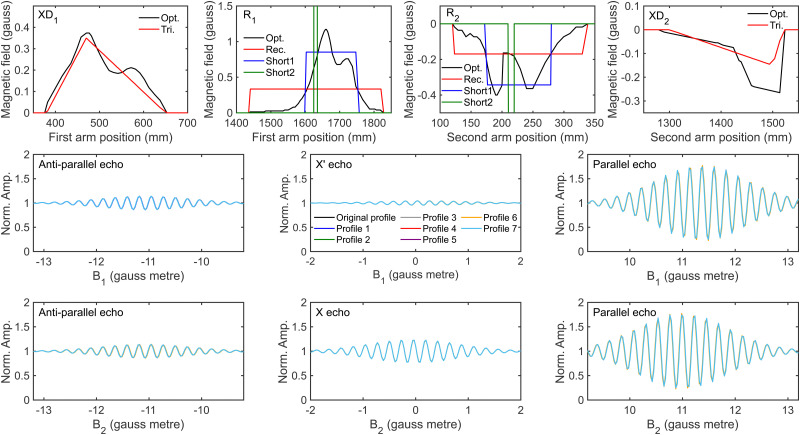
Comparison of the optimised magnetic field profile (black line) with the approximations made (coloured lines) for XD_1_ (top row, first panel), *R*_1_ (top row, second panel), *R*_2_ (top row, third panel) and XD_2_ (top row, fourth panel), as well as the simulated anti-parallel (*B*_1_ = −*B*_2_, second row, left panel), X′ (second row, middle panel) and parallel (*B*_1_ = *B*_2_, second row right panel) echoes obtained by propagating ^3^He through these different magnetic field profiles for a *B*_1_ scan when *B*_2_ = 11.2 gauss metre, and the simulated anti-parallel (*B*_1_ = −*B*_2_, bottom row, left panel), X (bottom row, middle panel) and parallel (*B*_1_ = *B*_2_, bottom row right panel) echoes obtained by propagating ^3^He through these different magnetic field profiles for a *B*_2_ scan when *B*_1_ = 11.2 gauss metre.

To do this, the optimised residual fields *R*_1_ and *R*_2_ were replaced with different length and different height rectangles, where the parameters were chosen to maintain the same field integral, and XD_1_ and XD_2_ were replaced with triangles which correctly reproduced the amplitudes of the X and X′ echoes respectively. The different approximations used for each of the non-ideal fields are compared with those that are in the optimised profile (black lines) in the top row of Fig. 10. These were then used to define alternative magnetic field profiles which combined the measured profiles for the solenoids and dipole fields with the approximate fields for *R*_1_ and *R*_2_, or XD_1_ and XD_2_, or both, with the details of which profile contained which approximation given in Table 3. As shown by the bottom two rows of Fig. 10, which presents the signals simulated for the different echoes for ^3^He scattering calculated using wave-functions propagated through these different profiles, the signals that are obtained from each profile are indistinguishable.

**Table 3:** The components of the different magnetic field profiles that were used in combination with the measured dipole and solenoid fields to simulate the signals presented in the bottom two rows of Fig. 10, and to obtain the scattering-matrix elements presented in Fig. 11. The definition of each component for the non-ideal field is presented in the corresponding panel in the top row of Fig. 10

Non-ideal field	Profile						
	1	2	3	4	5	6	7
*R*_1_	Rec.	Short1	Short2	Opt.	Short1 shifted +5 mm	Short1 shifted −5 mm	Short1
*R*_2_	Rec.	Short1	Short2	Opt.	Short1 shifted +5 mm	Short1 shifted −5 mm	Short1
XD_1_	Opt.	Opt.	Opt.	Tri.	Opt.	Opt.	Tri.
XD_2_	Opt.	Opt.	Opt.	Tri.	Opt.	Opt.	Tri.

**Fig. 11:**
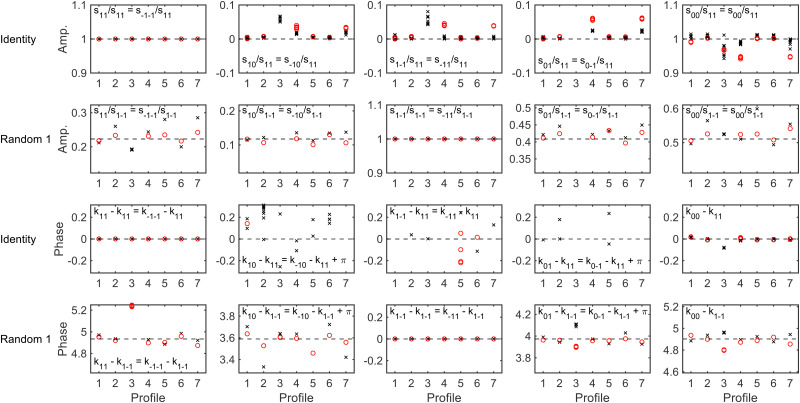
The (unique) amplitudes (top two rows) and phases (bottom two rows) of the scattering-matrix elements obtained by fitting the signals generated for an identity scattering-matrix (first and third row) and the first randomly generated scattering-matrix (second and fourth row) with 1% noise using the 50 μm expansion hexapole probabilities (black crosses) and the 250 μm shifted by 200 μm expansion (red circles) using the magnetic field profiles noted along the *x*-axis. The magnetic field profiles used in the propagation correspond to those defined in Table 3. The dashed horizontal lines show the value of the scattering-matrix elements used to simulate the data.

To determine whether these different profiles affect the ability to extract a scattering-matrix from the H_2_ data, fits were run to signals which included 1% noise for the identity and first randomly generated scattering-matrix using the wave-functions propagated through the magnetic field profiles given in Table 3. The results obtained for fitting the signal generated using an identity *S*-matrix are presented in the first and third rows of Fig. 11, with the top row presenting the scattering-matrix amplitudes, and the third the phases, and the amplitudes and phases obtained from fitting the signal simulated using the first randomly generated scattering-matrix are shown in the second and fourth rows, respectively. The values obtained for the ten fits with the minimum error are shown in each panel for the signal simulated using hexapole probabilities corresponding to a 50 μm expansion (black crosses) and 250 μm expansion shifted by 200 μm (red circles). Whilst fitting the data, the scattering-matrix elements, a background parameter, and the two parameters which can account for the difference in the hexapole probabilities were allowed to vary. Replacing the measured *R*_1_ and *R*_2_ fields with a rectangle which is the same length as the residual field (profile 1) or approximately half the length (profile 2) does not significantly affect the ability to extract the scattering-matrix values used in the simulations. Shifting this shorter profile by +5 mm (profile 5) or −5 mm (profile 6) also does not have a significant effect on the extraction of the *S*-matrix elements for the identity matrix (first and third row), although the deviation in the random scattering-matrix amplitudes is larger (second row). Changing the measured XD_1_ and XD_2_ fields to triangles (profiles 4 and 7) tends to have a larger effect, making the extracted scattering-matrix elements and those used to simulate the signals deviate more, but as a general rule the *S*-matrix amplitudes are still within 0.05 of those used in the simulations, and phases within 0.2. The largest deviations are seen in the case of profile 3, where the optimised *R*_1_ and *R*_2_ have been replaced by a rectangular residual field which is 10 times shorter and 10 times larger (to keep the same field integral). In some of the panels, the results for profile 3 cannot be seen due to the size of the deviations, where the amplitudes obtained from the fit can be up to 0.4 away from the value used to simulate the data, and the maximum difference in the phases being approximately 3, which effectively flips the phase of the oscillation of that component of the fit. Whilst these differences are unquestionably significant and would reduce the ability to extract reliable scattering-matrix elements from MMI measurements, magnetic field profile 3 is unrealistic given the measured magnetic field profiles and the possible uncertainties that are present in them. The remaining profiles are a more realistic representation of the possible uncertainties in the measured magnetic field profiles, although should still be considered to represent extreme cases, for example in profiles 5 and 6 the (rectangular) residual fields have been shifted by ±5 mm, whereas the uncertainties in the positions are more likely to be on the order of ±2 mm. Likewise, the length of *R*_1_ and *R*_2_ are unlikely to be wrong by a factor of 2 (which corresponds to approximately 10 cm), which is the change that was made between profiles 1 and 2, which the results show does not significantly change the value of the scattering-matrix elements obtained by fitting the simulated signals.

To summarise this section, it is not sufficient to just rely on the comparison between simulated and measured signals for ^3^He scattering to obtain the magnetic field profile, as all seven profiles considered here produced the same simulated ^3^He signals. Instead, it is essential to measure the magnetic field profile as accurately as possible as this provides a starting point for characterising the profiles of the beamline. Once this is obtained, it can be tweaked within the limits of what the uncertainties can realistically be, without affecting the ability to extract the scattering-matrix elements. This demonstrates that this procedure is robust, and the uncertainties in it do not restrict the analysis of the MMI signals to obtain reliable empirical *S*-matrices.

## Summary and conclusions

The magnetic molecular interferometer technique provides a powerful tool which can be used to obtain empirical scattering-matrices^28,29^ which can in turn be used to benchmark the accuracy of state-of-the-art theoretical models. However, both the experimental and analytical methods are complicated and any uncertainties in these would be transferred to uncertainties in the *S*-matrix parameters that are obtained from analysing the data. The current work has studied the effect of these uncertainties and the influence they have on the extracted values of the scattering-matrix.

To calculate the signal accurately requires knowledge of both the probabilities that the nine different *m*_*I*_, *m*_*J*_ states of H_2_ in *I* = 1, *J* = 1 are transmitted through the two hexapoles of the apparatus, and the magnetic field profile of the machine. The 81 hexapole probabilities can be obtained using semi-classical trajectory calculations, and the resulting probabilities can be described by a double Gaussian model. Whilst this is characterised by five parameters (plus an arbitrary normalisation constant), only two need to be allowed to vary to account for the uncertainty in the size and the position of the molecular beam expansion in the MMI apparatus. Freeing these two parameters in fits to simulated data does not hamper the ability to extract a scattering-matrix when there is a good signal to noise ratio, which corresponds to errors on the order of 1% of the maximum oscillation amplitude. Even with a higher signal to noise ratio which may prevent an *S*-matrix being reliably extracted from the data, the measurement can still be used to benchmark calculations by comparing signals calculated using theoretical results and those measured experimentally. Whilst this is a less good test than being able to compare empirical and calculated *S*-matrix values directly, the oscillation curves measured experimentally are interference patterns, and therefore the comparison between the measurement and the calculated signal is still sensitive to both the amplitudes and phases of the *S*-matrix elements.

To determine the magnetic field profile, the *X*, *Y* and *Z* components of the field in the beamline were measured as accurately as possible using commercially available gauss meters.^39^ Whilst these measurements provide a starting point, ^3^He measurements are used to adjust the non-ideal fields in the magnetic field profile, *i.e.*, the magnitude of the residual fields in both arms of the machine (*R*_1_ and *R*_2_) and the *X* and *X*′ components of the dipoles (XD_1_ and XD_2_), until the simulated signals agree with those determined experimentally at a range of nozzle temperatures. The residual fields are obtained by comparing the parallel^50^ (*B*_1_ = *B*_2_) and anti-parallel^50^ (*B*_1_ = −*B*_2_) echoes between the measured and calculated profiles, and the *X* and *X*′ components of the dipoles determined using the intensity of the X and X′ echoes.^33^ The profiles are then validated by comparing with data measured where the non-scanned magnetic field was fixed at 0 gauss metre. As has been demonstrated, the same simulated signals for ^3^He can be produced using wave-functions that have been propagated through several different magnetic field profiles which share the same field integral values. Fitting simulated H_2_ scattering signals with wave-functions propagated through each of these different profiles shows that the *S*-matrix elements used to simulate the signals can be obtained when the magnetic field profile is similar to the measured profile, and only adjusted within what could be a reasonable measurement error. Changing the profile more than this can produce the same simulated signals for ^3^He scattering but results in significant errors in *S*-matrix elements extracted from H_2_ scattering experiments, demonstrating that the combination of measuring the magnetic field profile and ^3^He simulations and experiments are required to obtain a profile that can then be used to analyse H_2_ MMI measurements, and that using either individually does not produce a magnetic field profile for the beamline with sufficient accuracy. The relative insensitivity of the fitting procedure to the details of the non-ideal fields demonstrates the robustness of the methods used for obtaining empirical scattering-matrices.

## Data availability

Data for this article, including the experimental data, simulations, and analysis, as well as the codes used to generate the figures, are available on Zenodo at https://doi.org/10.5281/zenodo.11517850.

## Conflicts of interest

There are no conflicts to declare.

## Supplementary Material

CP-026-D4CP01785D-s001
