# Supplementary material for: Characterisation of magnetic atomic and molecular beamlines for the extraction of empirical scattering-matrices
Source: Phys Chem Chem Phys. 2024 Jun 11;26(29):19630–45. doi: 10.1039/d4cp01785d (PMC11267877; doi:10.1039/d4cp01785d)
Supplement: CP-026-D4CP01785D-s001 [file CP-026-D4CP01785D-s001.pdf]

Electronic Supplementary Material (ESI) for Physical Chemistry Chemical Physics.
This journal is © the Owner Societies 2024

Supplementary Information for

Characterisation of magnetic atomic and molecular beamlines for the extraction of empirical scattering-matrices

Helen Chadwick

Effect of the non-ideal fields on magnetic molecular interferometry signals.

The magnetic field profile for the first arm of an ideal magnetic molecular interferometer (MMI) beamline would consist of an initial dipole field along Z (see Fig. 1 for the definition of the axes), followed by a region of zero field, after which there is a (rectangular) solenoid field directed along (-)X, which is again followed by a region of zero field before the surface. The magnetic fields in the second arm can be considered a mirror of the first, consisting of an initial zero field region followed by a (rectangular) solenoid field directed along (-)X', after which is another zero field region before a final dipole field along the (-)Z' direction. As shown in Fig. 8, the beamline of the magnetic molecular interferometer apparatus has additional small 'non-ideal' fields, in the form of X and X' fields at the end of the first and start of the second dipole (referred to as XD_1 and XD_2 respectively), and residual fields along X and X' at the entrance and exit of the scattering chamber (referred to as R_1 and R_2 respectively), which also need to be characterised. These non-ideal fields can affect the measured MMI signals, as will be demonstrated using simulated signals for the scattering of ^3He below. The results shown here provide the justification for the methodology used to optimise these fields presented in the main manuscript.

Figure S1 shows calculated signals for ^3He scattering simulated for scanning the solenoid field in the first arm, B_1, when the solenoid field in the second arm, B_2, is held at a constant field of 11.2 gauss metre, where only R_1 has been changed (top row), only R_2 has been changed (second row), R_1 and R_2 have been changed simultaneously but $R_1 - R_2 = R_-$ is constant (third row), R_1 and R_2 have been changed simultaneously but $R_1 + R_2 = R_+$ is constant (fourth row), only XD_1 has been changed (fifth row) and only XD_2 has been changed (sixth row). In each signal there are three spin-echoes which correspond to the anti-parallel[1] ($B_1 = -B_2$, left panel), parallel[1] ($B_1 = B_2$, right panel) and X'[2] echoes which have been discussed previously. Equivalent simulations are presented in Fig. S2 for B_2 scans when B_1 is held at a constant field of 11.2 gauss metre, with the middle column in this case corresponding to the X echo[2]. Focussing initially on the effect of changing R_1 and R_2 on the parallel and anti-parallel echoes (left and right column respectively of Figs. S1 and S2), it can be seen that changing only R_1 or R_2 (top two rows) shifts the position of the echoes. However, changing R_1 and R_2 simultaneously but keeping $R_1 - R_2$ fixed at R_- (third row) does not change the simulated signal for the parallel echo (right column) and only changes the phase of the anti-parallel echo (left column). The opposite is true if R_1 and R_2 are varied whilst $R_1 + R_2$ is a constant value of R_+ (fourth row), with the phase of the parallel echo (right column) changing whereas the anti-parallel echo (left column) is unaffected.

The results for how R_1 and R_2 affect the parallel and anti-parallel echoes can be understood by considering the expression for the signal which has been derived previously for the propagation of ^3He through rectangular solenoid fields with magnetic field values B_1' and B_2' (note the prime on these values which distinguish these from the integral magnetic field values that are used throughout the manuscript) which is given by[1]

$$\text{sig} = \frac{1}{2} + \frac{1}{2}\cos^2\frac{\theta_B}{2}\cos((B_1{}' + B_2{}')\gamma t) - \frac{1}{2}\sin^2\frac{\theta_B}{2}\cos((B_1{}' - B_2{}')\gamma t) \tag{S1}$$

where γ is the gyromagnetic ratio for ^3He, θ_B is the angle between B_1 and B_2 (equivalently the X and X' directions) and t is the time the ^3He atoms spend in the solenoid field (which are assumed to be rectangles of the same length). The first non-constant term in the expression corresponds to the anti-parallel echo ($B_1 = -B_2$), and the second to the parallel echo ($B_1 = B_2$). Whilst this expression was derived without considering the effect of residual fields, it can be modified by considering B_1' to be the total field in the first arm (i.e., $B_1' + R_1'$) and B_2' to be the total field in the second arm (i.e., $B_2' + R_2'$) where R_1' and R_2' are rectangular residual fields which are the same length as the solenoid fields. Modifying the expression in this way allows Eq. (S1) to be rewritten as

$$\text{sig} = \frac{1}{2} + \frac{1}{2}\cos^2\frac{\theta_B}{2}\cos((B_1' + B_2' + R_1' + R_2')\gamma t) - \frac{1}{2}\sin^2\frac{\theta_B}{2}\cos((B_1' - B_2' + R_1' - R_2')\gamma t) \quad \text{(S2)}$$

It follows that adding a residual field, R_1', in the first arm or R_2' in the second arm, leads to a shift in the echo positions as shown in the top two rows of Figs. S1 and S2. However, changing R_1' and R_2' but keeping R_1' - R_2' fixed only affects the first term but not the second for a given value of B_1' and B_2', which means that the phase of the anti-parallel echo changes, but the parallel echo is unaffected, as shown by the left and right columns of the third row of the two figures. Finally, changing R_1' and R_2' but keeping R_1' + R_2' constant means that the first term does not change leaving the anti-parallel echo the same for a given value of B_1' and B_2', but the second term does change, which changes the phase of the parallel echo, as is shown in the left and right columns of the fourth row of Figs. S1 and S2.

Making the substitutions $\cos(N \pm M) = \cos N \cos M \mp \sin N \sin M$ where $N = (B_1' + R_1{}')\gamma t$ and $M = (B_2' + R_2{}')\gamma t$, allows Eq. (S2) to be rewritten as

$$\text{sig} = \frac{1}{2} + \frac{1}{2}\cos((B_1' + R_1{}')\gamma t)\cos((B_2' + R_2{}')\gamma t)\cos(\theta_B) - \frac{1}{2}\sin((B_1' + R_1{}')\gamma t)\sin((B_2' + R_2{}')\gamma t) \quad \text{(S3)}$$

Rewriting the expression for the signal in this form reveals another interesting effect that the residual fields R_1 and R_2 can have on the signal when either B_1 is scanned and B_2 is off (B_2 = 0 gauss metre) or vice-versa. Considering the case of a B_1 scan around B_2 = 0 gauss metre and therefore setting the value of B_2' to be zero in Eq. (S3), it immediately follows that the amplitude of the two terms in Eq. (S3) depend on the value of R_2', which means that the amplitude of the resulting signal will depend on the value of R_2'. As the fields in the first arm of the apparatus (subscript 1) and second arm of the apparatus (subscript 2) are interchangeable in Eq. (S3) (i.e., switching subscripts 1 and 2 on every term in the expression gives the same expression for the signal), the same is true for a B_2 scan when B_1 is zero, i.e., that the amplitude of the oscillation would depend on the value of R_1. This effect is shown in the simulated signals presented in the top (bottom) panel of Fig. S3 for a B_1 (B_2) scan where B_2 (B_1) is fixed at 0 gauss metre, for different values of R_1 and R_2. However, it is interesting to note that the changing amplitude is not observed in the echo data presented in Figs. S1 and S2, as the larger value of B_2 (B_1) in the case of a B_1 (B_2) scan has effectively already randomised the direction of the spin projection due to the velocity spread in the beam. This can be seen in Fig. S3 as the oscillations in the signal have already decayed by ±3 gauss metre, meaning the spin projection is already essentially randomised if B_1 or B_2 are fixed at values of > 3 gauss metre. Adding a small residual field does not change this, so the amplitude of the signal in the case of the echoes does not change as R_1 or R_2 is changed.

The X' and X echoes shown in the middle column of Figs. S1 and S2 respectively are not captured by Eqs. (S1) to (S3), as they arise due to the XD_1 and XD_2 components of the magnetic field[2], which were not considered in the derivation of Eq. (S1)[1]. As shown by the bottom two rows of the figures, the most significant effect of changing XD_1 from its initial value (XD_1 = 1, black solid line) to either double (XD_1 = 2, blue dotted line) or half its initial value (XD_1 = 0.5, red dashed line) is that it changes the magnitude of the X echo (middle panel, fifth row, Fig. S2). Analogously, when XD_2 is changed the most significant effect it has is to change the magnitude of the X' echo (middle panel, bottom row, Fig. S1), but this is less clear in the current work due to the small magnitude of that echo initially. The effect of changing XD_1 and XD_2 in the same way on the anti-parallel (bottom two rows, left column) and parallel (bottom two rows, right column) echoes is significantly less. As shown in the middle panel of the second row of Fig. S1, the X' echo is independent of R_2, and in the middle panel of the top row of Fig. S2, the X echo is independent of R_1. This is due to the mechanism through which these echoes arise, which has been discussed previously[2].

References

1 I. Litvin, Y. Alkoby, O. Godsi, G. Alexandrowicz and T. Maniv, *Results Phys.*, 2019, **12**, 381–391.

2 H. Chadwick, J. T. Cantin, Y. Alkoby and G. Alexandrowicz, *J. Phys. Condens. Matter*, 2022, **34**, 345901.

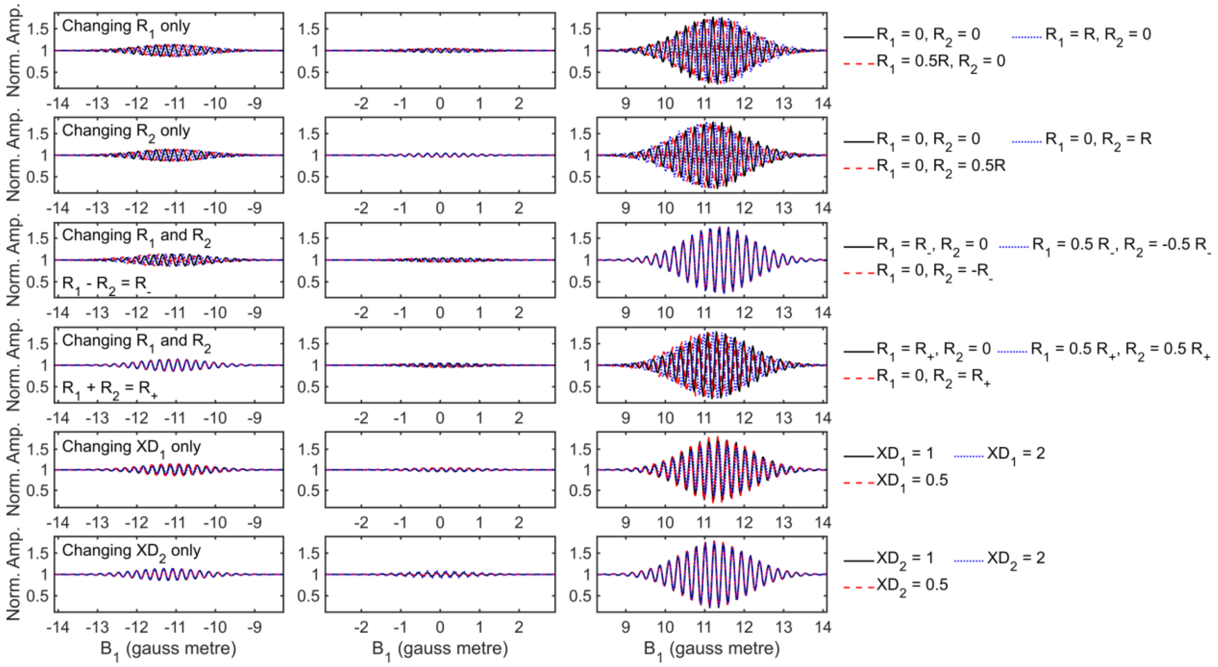

Figure S1. The effect of changing the non-ideal fields in the measured magnetic field profile on simulated MMI signals for ^{3}He scattering for the anti-parallel (left column), X' (middle column) and parallel (third column) echoes obtained when scanning B_1 for B_2 = 11.2 gauss metre. The changes made to the measured profile correspond to changing R_1 only between 0 and a fixed (arbitrary) value, R (top row), to changing R_2 only between 0 and the same fixed arbitrary value, R (second row), to changing R_1 and R_2 only but keeping $R_1 - R_2$ (= R_-) constant (third row), to changing R_1 and R_2 only but keeping $R_1 + R_2$ constant (fourth row), to changing XD_1 only (fifth row) and to changing XD_2 only (sixth row).

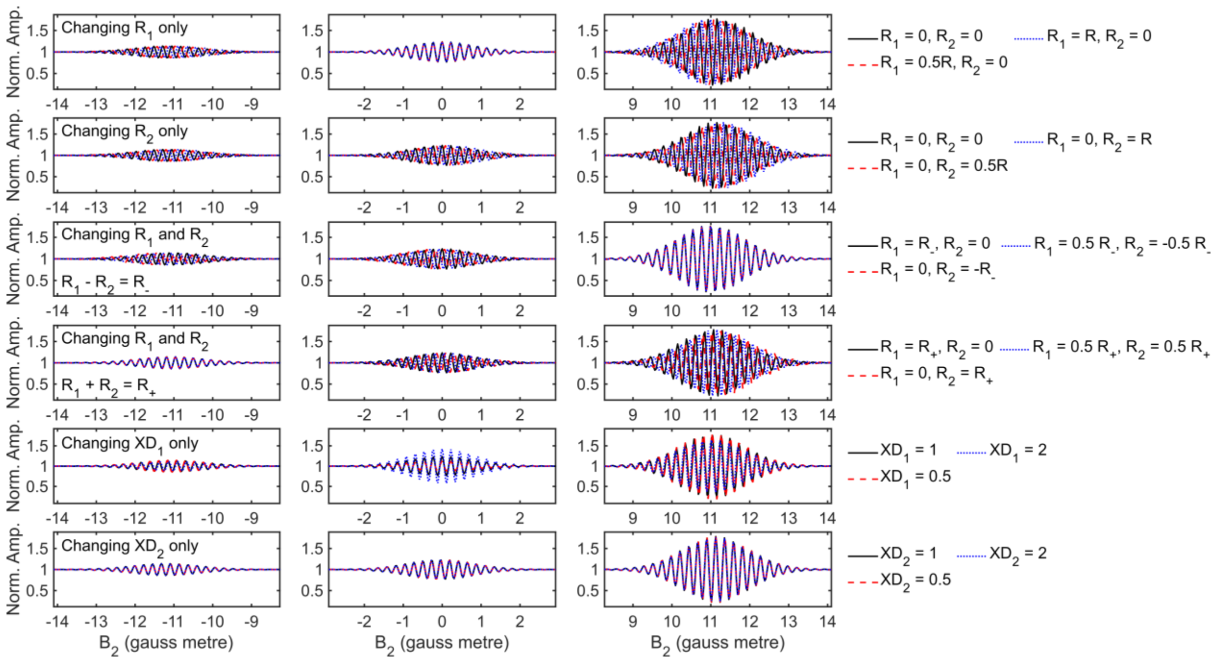

Figure S2. The effect of changing the non-ideal fields in the measured magnetic field profile on simulated MMI signals for ^{3}He scattering for the anti-parallel (left column), X (middle column) and parallel (third column) echoes obtained when scanning B_2 for B_1 = 11.2 gauss metre. The changes made to the measured profile correspond to changing R_1 only between 0 and a fixed (arbitrary) value, R (top row), to changing R_2 only between 0 and the same fixed arbitrary value, R (second row), to changing R_1 and R_2 only but keeping $R_1 – R_2$ (= R_-) constant (third row), to changing R_1 and R_2 only but keeping $R_1 + R_2$ (= R_+) constant (fourth row), to changing XD_1 only (fifth row) and to changing XD_2 only (sixth row).

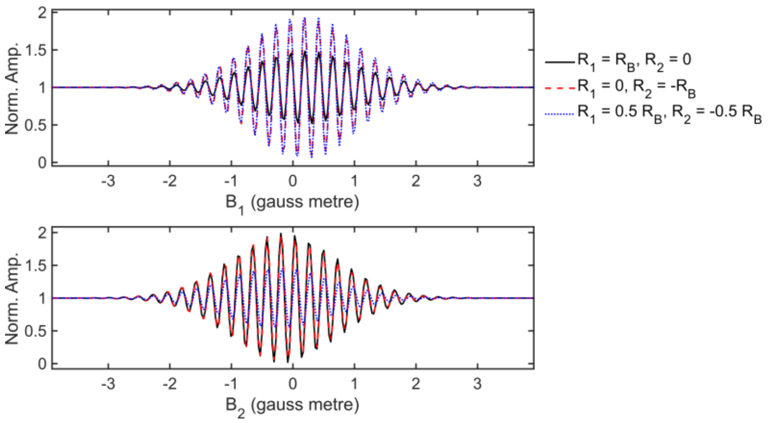

Figure S3. Simulated signals for ^{3}He scattering from Cu(111) for B_1 scans when B_2 = 0 gauss metre for fixed values of $R_1 - R_2 = R_-$ for $R_1 = R_-$ and $R_2 = 0$ (black solid line), $R_1 = 0$ and $R_2 = -R_-$ (red dashed line) and $R_1 = 0.5R_-$ and $R_2 = -0.5R_-$ (blue dotted line) (top panel) and B_2 scans when B_1 = 0 gauss metre for the same values of R_1 and R_2 (bottom panel).

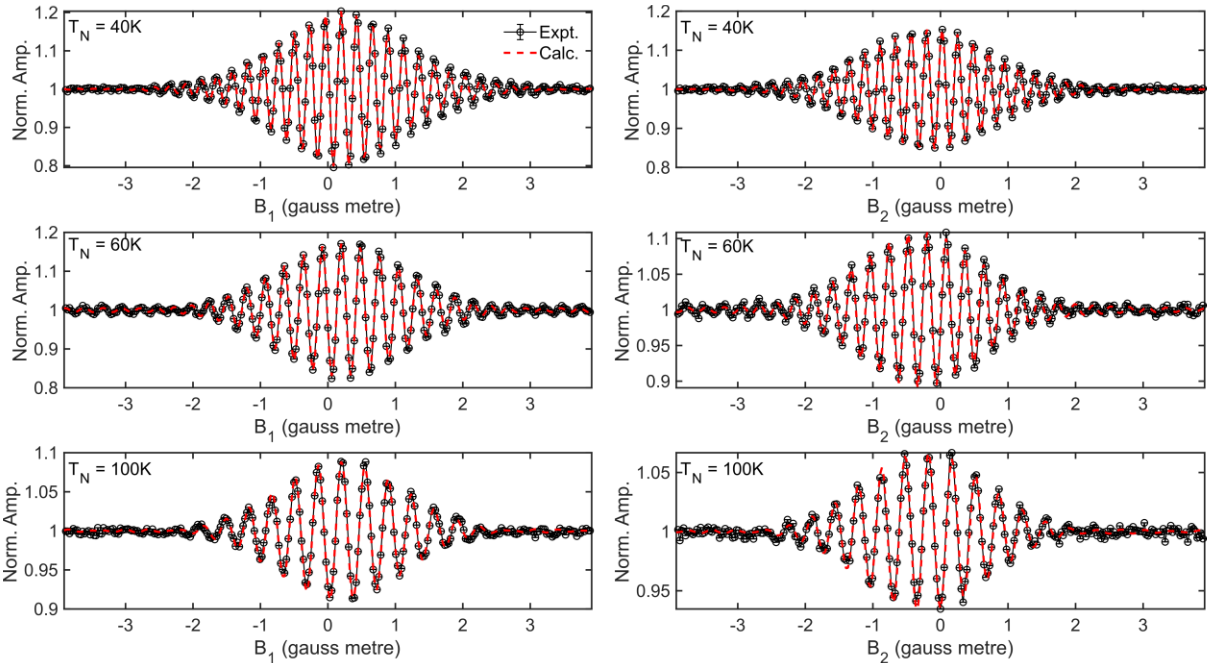

Figure S4. Comparison of the measured [3]He signals (black solid line) with the calculated signals (red dashed line) for B_1 scans when B_2 = 0 gauss metre (left column) and B_2 scans when B_1 = 0 gauss metre (right column) at nozzle temperatures (T_N) of 40K (top row), 60K (middle row) and 100K (bottom row).
